# Immunosuppressive capacity of circulating MDSC predicts response to immune checkpoint inhibitors in melanoma patients

**DOI:** 10.3389/fimmu.2023.1065767

**Published:** 2023-02-13

**Authors:** Vera Petrova, Christopher Groth, Rebekka Bitsch, Ihor Arkhypov, Sonja C. S. Simon, Svetlana Hetjens, Verena Müller, Jochen Utikal, Viktor Umansky

**Affiliations:** ^1^ Skin Cancer Unit, German Cancer Research Center (DKFZ), Heidelberg, Germany; ^2^ Department of Dermatology, Venereology and Allergology, University Medical Center Mannheim, Ruprecht-Karl University of Heidelberg, Mannheim, Germany; ^3^ DKFZ-Hector Cancer Institute at the University Medical Centre Mannheim, Mannheim, Germany; ^4^ Mannheim Institute for Innate Immunoscience (MI3), Medical Faculty Mannheim, University of Heidelberg, Mannheim, Germany; ^5^ Department of Medical Statistics and Biomathematics, University Medical Center Mannheim, Medical Faculty Mannheim of the University of Heidelberg, Mannheim, Germany

**Keywords:** MDSC, melanoma, immunosuppression, immune checkpoint inhibitors, cytokines

## Abstract

**Purpose:**

Although the treatment of advanced melanoma patients with immune checkpoint inhibitors (ICI) significantly increased the therapeutic efficiency, many patients remain resistant to ICI that could be due to immunosuppression mediated by myeloid-derived suppressor cells (MDSC). These cells are enriched and activated in melanoma patients and could be considered as therapeutic targets. Here we studied dynamic changes in immunosuppressive pattern and activity of circulating MDSC from melanoma patients treated with ICI.

**Experimental design:**

MDSC frequency, immunosuppressive markers and function were evaluated in freshly isolated peripheral blood mononuclear cells (PBMC) from 29 melanoma patients receiving ICI. Blood samples were taken prior and during the treatment and analyzed by flow cytometry and bio-plex assay.

**Results:**

MDSC frequency was significantly increased before the therapy and through three months of treatment in non-responders as compared to responders. Prior to the ICI therapy, MDSC from non-responders displayed high levels of immunosuppression measured by the inhibition of T cell proliferation assay, whereas MDSC from responding patients failed to inhibit T cells. Patients without visible metastasis were characterized by the absence of MDSC immunosuppressive activity during the ICI treatment. Moreover, non-responders showed significantly higher IL-6 and IL-8 concentrations before therapy and after the first ICI application as compared to responders.

**Conclusions:**

Our findings highlight the role of MDSC during melanoma progression and suggest that frequency and immunosuppressive activity of circulating MDSC before and during the ICI treatment of melanoma patients could be used as biomarkers of response to ICI therapy.

## Introduction

Malignant melanoma is characterized by high mutational burden and increased immunogenicity (1, 2) but also by a profound immunosuppression (3). The latter represents one of the major reasons for poor therapy responses (4, 5). The application of immune checkpoint inhibitors (ICI), including antibodies against programmed cell death protein 1 (PD-1) and cytotoxic T-lymphocyte protein 4 (CTLA-4) significantly increased survival and response rates for advanced melanoma patients (6). However, many patients fail to respond to ICI. In particular, only 20% melanoma patients respond to anti-CTLA-4 treatment, 30-40% respond to anti–PD-1 antibodies and 58% show clinical response to the combination therapy with these antibodies (7). Since the rate of the non-responsiveness to ICI is high, there is an urgent need to understand the underlying mechanisms of immunosuppression and to find biomarkers predicting clinical responses to ICI.

One of the major players in the immunosuppressive tumor microenvironment (TME) are myeloid-derived suppressor cells (MDSC). This heterogeneous population of myeloid cells emerges under chronic inflammatory conditions typical for cancer and is characterized by a strong ability to suppress anti-tumor T and NK cells *via* different mechanisms (8–10). In humans, three MDSC subsets have been described: CD33^+^HLA-DR^low/−^CD14^+^CD66b^−^ monocytic (M-MDSC) and CD33^dim^HLA-DR^low/−^CD14^−^CD66b^+^Lin^−^ polymorphonuclear (PMN-MDSC) that are strongly immunosuppressive as well as CD33^dim^HLA-DR^low/−^CD66b^−^Lin^−^ early-stage MDSC (e-MDSC), which fail to show immunosuppressive function (11, 12). Cells with typical MDSC phenotype are also present in healthy individuals in much smaller numbers and called non-immunosuppressive MDSC counterparts (12).

Generation, expansion, and recruitment of MDSC to the TME is influenced by cytokines, chemokines and growth factors produced by melanoma and host cells such as interleukin (IL)-1β, IL-6, IL-8, vascular endothelial growth factor (VEGF), macrophage colony-stimulating factor (M-CSF) and granulocyte-macrophage colony-stimulating factor (GM-CSF) (9, 13). MDSC immunosuppressive function is stimulated by IL-1β, IL-6, IL-8, IL-4, IL-13, interferon γ (IFN-γ) and prostaglandin E2 (PGE2) (13).

It has been demonstrated that MDSC strongly express PD-L1, leading to T cell suppression of in the circulation and TME (14). MDSC display ectonucleoside triphosphate diphosphohydrolase 1 (CD39) and ectonucleotidase (CD73) expression, catalyzing the conversion of extracellular ATP into adenosine that inhibits effector T cell functions (15). The combination of CD39/CD73 targeting and ICI was reported to stimulate anti-tumor immunity in preclinical models (16). Moreover, MDSC produce reactive oxygen species (ROS) and nitric oxide (NO), which cause T cell anergy by the down-regulation of TCR ζ-chain expression (17, 18). All these mechanisms of immunosuppression mediated by MDSC support the tumor escape and reduce response to different melanoma therapies, including the ICI treatment. Even though the role of MDSC is well investigated, ICI-related changes in freshly isolated circulating MDSC from melanoma patients are not sufficiently studied, especially in patients with no evidence of disease (non-metastatic patients), receiving ICI in adjuvant setting. Here we analyzed the characteristics and function of MDSC as well as MDSC-related inflammatory mediators in the peripheral blood of 29 melanoma patients before and during ICI treatment. We found that elevated baseline frequency of MDSC, their high immunosuppressive activity as well as increased baseline levels of IL-6 and IL-8 are associated with unfavorable response to ICI treatment in metastatic patients. In contrast, MDSC from responding metastatic patients tend to lose their ability to suppress T cell functions under the ICI treatment. We suggest that the combination of ICI and MDSC targeting could improve the efficiency of melanoma immunotherapy.

## Materials and methods

### Patients and healthy donors

**Figure 1:**
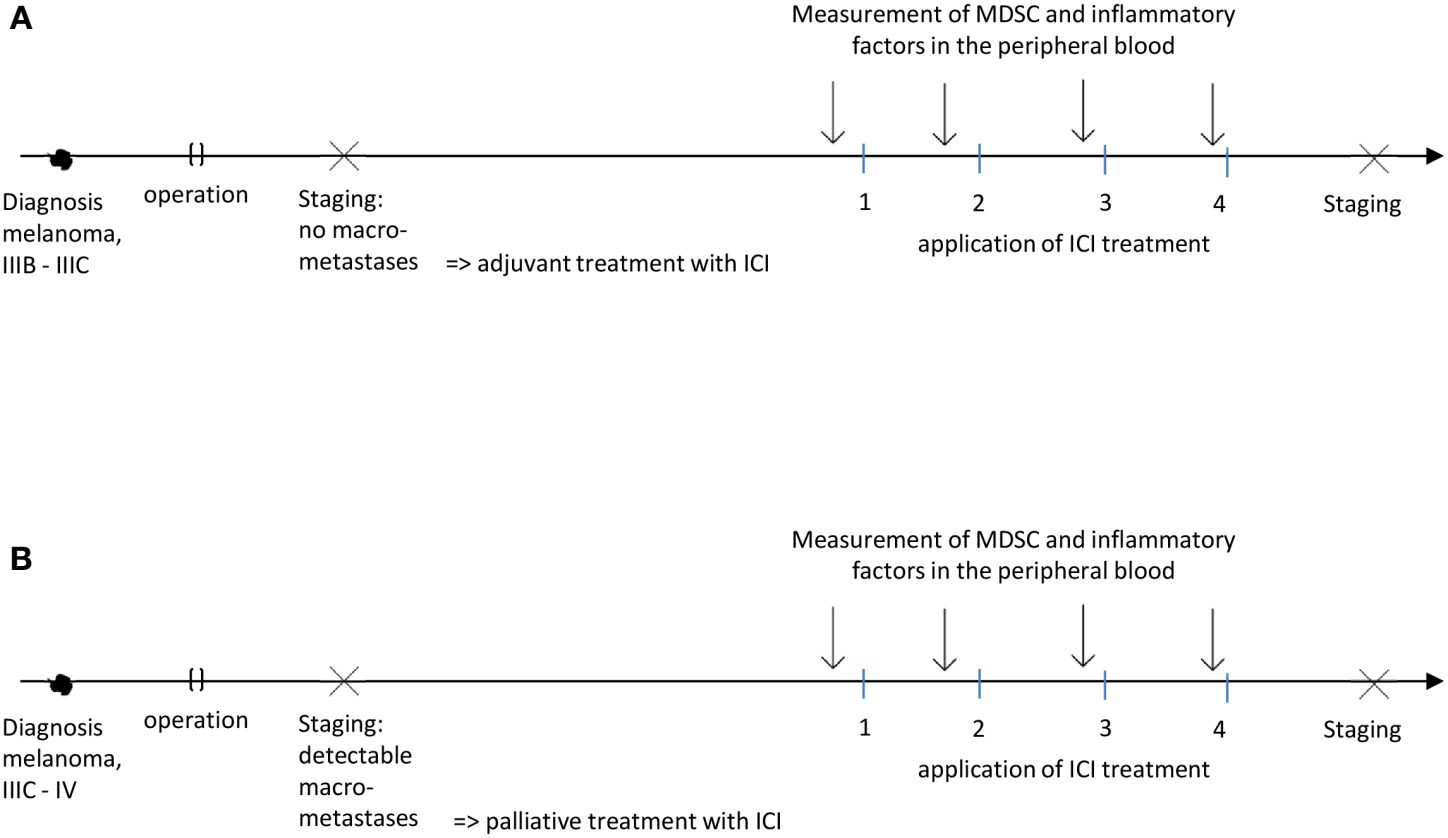
Schematic representation of adjuvant **(A)** or palliative **(B)** treatment settings (patients with no evidence of disease and those with distant metastases respectively).

For this study, peripheral blood samples were collected from 19 metastatic and 10 non-metastatic melanoma patients receiving ICI at the Skin Cancer Center (University Medical Center Mannheim, Germany). Metastatic patients received ICI as a palliative treatment and non-metastatic patients as an adjuvant therapy (**Figure 1**). To simplify the narration, patients receiving ICI in palliative or adjuvant regimen will be called metastatic and non-metastatic patients respectively. This study was conducted in accordance with the Declaration of Helsinki and approved by the local Ethics Committee (2010-318N-MA). Peripheral blood from 10 age- and gender-matched healthy donors (HD) without indications of immune-related diseases was obtained according to the Ethics Committee approval (2010-318N-MA) and used as controls. The collection of samples and clinical data was performed after written informed consent.

### Clinical data

Metastatic patients with unresectable stage IIIC-IV melanoma (according to the AJCC 2017 classification) were treated with nivolumab, 480 mg (fixed dose) every 4 weeks or pembrolizumab, 200 mg, every 3 weeks. Metastatic patients with presence of brain metastasis were treated every 3 weeks with a combination of nivolumab, 1 mg/kg body weight, and ipilimumab, 3 mg/kg body weight. One patient from the metastatic group deceased before the therapy start. Non-metastatic melanoma patients with stage IIIB-IIID without current evidence of disease were treated with nivolumab, 3mg/kg body weight, every 2 weeks or pembrolizumab, 200 mg, every 3 weeks. In this group, 3 patients received nivolumab, 480 mg, every 4 weeks before the new dosage for adjuvant treatment was approved. All recruited patients received no immunotherapy before the study onset. Treatment efficacy was assessed by contrast-enhanced computed tomography (CT), magnetic resonance imaging (MRI), or positron emission tomography CT (PET-CT) based on the Immunotherapy Response Evaluation Criteria in Solid Tumors (iRECIST) 3 months after the first administration of ICI. Based on the response at this time point, metastatic patients were divided into responders showing complete response (CR), partial response (PR) or stable disease (SD) and non-responders (progressive disease, PD). Patient characteristics are summarized in **Table 1**.

**Table 1:** Clinical characteristics of melanoma patients treated with ICI in palliative and adjuvant settings.

	Palliative treatment (n=19)	Adjuvant treatment (n=10)	Healthy donors (n=10)
Median age, years (range)	64 (41-84)	67 (34-84)	60 (38-73)
Sex, n			
Male	13	8	5
Female	6	2	5
AJCC stage, n			
IIIB	0	6	
IIIC	3	3	
IIID	0	1	
IV	16	0	
Primary melanoma site, n			
Cutaneous	13	10	
Uveal	1	0	
Unknown	5	0	
Therapy, n			
Nivolumab	12	9	
Pembrolizumab	1	1	
Nivolumab + ipilimumab	5	0	
Deceased before therapy start	1	0	
Therapy outcome, n			
Responder	12		
Complete response	1		
Partial response	8		
Stable disease	3		
Non-responder (progressive disease)	6		

### Analysis of peripheral blood samples

Peripheral blood was collected from melanoma patients before (baseline) and after the 1^st^, 2^nd^, and 3^rd^ application of ICI with trisodium citrate as an anticoagulating agent. Peripheral blood mononuclear cells (PBMC) were isolated using density gradient centrifugation with Biocoll (Biochrom) and applied for flow cytometry and cell sorting. After the PBMC removal, plasma was collected and stored at -80°C.

### Flow cytometry

Freshly prepared PBMC were treated with FcR Blocking Reagent (130-111-568, Miltenyi Biotec) according to the manufacturer’s protocol and stained with fixable viability dye 700 (BD Biosciences) followed by the incubation with monoclonal antibodies (mAbs) for 30 min at 4°C. The following fluorescently labeled mAbs were used for the surface staining: CD66b-PerCPCy5.5 (clone G10F5), CD14-APCCy7 (clone MФP9), HLA-DR-V500 (clone G46-6), lineage cocktail (LIN) (CD3/19/20/56)-APC, CD33-PE-Cy7 (clone P67.6), CD39-FITC (clone TU66), PD-L1-BV421 (clone MIH1, all from BD Biosciences) and CD73-BV605 (clone AD2, Biolegend). Intracellular ROS and NO were detected using hROS Detection Kit (Cell Technology) and diaminofluorescein-FM diacetate (Cayman Chemical) according to the manufacturer’s instructions. Acquisition was performed by 10-color flow cytometry using BD FACSLyric with FACSuite software (BD Biosciences). FlowJo V 10 software (BD Biosciences) was used to analyze at least 10^6^ events. Positive surface markers were gated according to the fluorescence minus one (FMO) control.

### Inhibition of T cell proliferation assay

Immunosuppressive activity of MDSC during the ICI treatment was evaluated according to the standardized Mye-EUNITER protocol (12). Briefly, CD3^+^ T cells were isolated from PBMC by magnetic-activated cell sorting (MACS, Miltenyi Biotec) according to the manufacturer’s protocol. CD3 depleted PBMC were sorted for HLA-DR^-^/CD33^high^ M-MDSC and HLA-DR^-^/CD33^dim^/CD66b^+^/LIN^-^ PMN-MDSC. CD3 T cells were labeled with 20 µM cell proliferation dye eFluor 450 (CP-Dye405, eBioscience) and were cultured alone or with sorted PMN- or M-MDSC (T cells:MDSC ratio = 1:1) in a 96 well round bottom plate (Sarstedt) in L-lysine and L-arginine low RPMI-1640 medium (Thermo Fisher Scientific) supplemented with 100 IU/mL penicillin, 100 mg/mL streptomycin and 10% (v/v) FCS at 37°C. The plate was precoated for 3 hours with CD3 (clone OKT-3, eBioscience) and CD28 antibodies (clone CD28.2, Beckman Coulter). The proliferation of CD8^+^ T cells was assessed after 96 h of co-culture by measuring CPDye405 dilution at the BD FACSLyric™ flow cytometer.

### Bio-Plex assay

Concentrations of cytokines and chemokines in the serum of melanoma patients and HD were measured by the Bio-Plex Pro Human Cytokine 27-plex Assay (Bio-Rad) using the manufacturer’s protocol. Acquisition and data analysis were performed by bio-plex Manager™.

### Statistical analysis

Statistical analysis was performed using the GraphPad Prism software (Version 8.1.2). Data showing a Gaussian distribution were compared with the unpaired two-tailed Student’s t test and not normally distributed data with Mann-Whitney test. Mixed-effects analysis with multiple comparisons was used to compare treatment groups and to investigate dynamic changes during the treatment. Survival curves were generated using the Kaplan-Meier method, and the statistical comparison was done by the log rank (Mantel-Cox) test.

## Results

### Patient characteristics

29 melanoma patients receiving ICI and 10 HD gave informed consent to participate in a prospective clinical study (**Table 1**). In the adjuvant treatment group, eight patients remained relapse-free (80%) and two patients (20%) developed new metastases after the first staging. In the palliative treatment group one patient showed CR (6%), eight patients have PR (44%) and three patients showed SD (17%). Six individuals who showed PD (33%) were classified as non-responders. One patient died shortly before the therapy start.

### Immunosuppressive pattern and function of MDSC at the baseline

**Figure 2:**
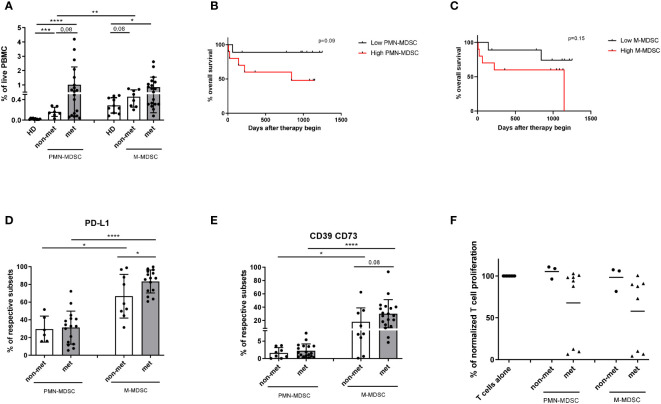
Characteristics of PMN- and M-MDSC from melanoma patients before ICI therapy. PBMCs were isolated from the peripheral blood of melanoma patients and HD. MDSC and their counterparts in HD were assessed by flow cytometry. **(A)** The results in metastatic (n=19) and non-metastatic (n=8) patients as well as their counterparts in HD (n=10) are presented as the percentage of HLA-DR^low/−^CD33^dim^CD66b^+^Lin^−^ PMN- and HLA-DR^low/−^CD33^high^CD14^+^ M-MDSC among live PBMC. **(B)** OS of metastatic melanoma patients with high (>0.54% of live PBMC; n=10) and low (<0.54%; n=9) PMN-MDSC frequencies at the baseline is shown as a Kaplan-Meier curve. **(C)** OS of metastatic melanoma patients with high (>0.73%; n=10) and low (<0.73%; n=9) M-MDSC frequencies at the baseline is shown as a Kaplan-Meier curve. **(D, E)** Expression of PD-L1 and ectoenzymes CD39 and CD73 on PMN- and M-MDSC from metastatic and non-metastatic patients was shown as the percentage of PD-L1^+^ cells **(D)** or CD39^+^CD73^+^ cells **(E)** among the respective MDSC subset. **(F)** Immunosuppressive capacity of PMN- and M-MDSC was determined upon the co-culture with activated CD3 T cells labeled with CP-Dye405. After 96 h of incubation, T cell proliferation was assessed by CP-Dye405 dilution measured by flow cytometry. Cumulative data for T cell proliferation are presented as the percentage of divided T cells normalized (norm.) to the respective control of stimulated T cells alone (n=3-8). *P < 0.05, **P < 0.01, ***P < 0.001, ****P < 0.0001.

First, we analyzed CD33^dim^HLA-DR^low/−^CD66b^+^Lin^−^ PMN- and CD33^+^HLA-DR^low/−^CD14^+^ M-MDSC in PBMC from melanoma patients and their non-suppressive MDSC counterparts from HD by flow cytometry. The gating strategy is shown in **Supplementary Figure 1**. We found a significant increase in the frequency of both PMN- and M-MDSC in metastatic melanoma patients (palliative therapy setting) and patients without metastases (adjuvant setting) as compared to their counterparts in HD (**Figure 2A**). The maximum of PMN-MDSC frequency was observed in metastatic patients with advanced disease (**Figure 2A**). To investigate a possible association between the frequency of circulating PMN- and M-MDSC within PBMC and the overall survival (OS) and progression-free survival (PFS) of melanoma patients, we distributed metastatic patients in the groups with low and high frequencies of PMN- and M-MDSC, using their median values (0.54% and 0.73% of live PBMC respectively) as a cutoff (19). We demonstrated that a high frequency of PMN-MDSC before therapy begin (>0.54%) was associated with the tendency for reduced OS and PFS (**Figure 2B**, **Supplementary Figure 2A**). High M-MDSC frequency (>0.73%) also showed a tendency to correlate with decreased OS (**Figure 2C**, **Supplementary Figure 2B**).

Next, we investigated an immunosuppressive pattern of different MDSC subsets in both metastatic and non-metastatic patients at baseline. Metastatic melanoma patients showed elevated frequencies of PD-L1^+^ M-MDSC as compared to non-metastatic patients (**Figure 2D**). Furthermore, the frequency M-MDSC expressing PD-L1 was higher that of PMN-MDSC (**Figure 2D**). However, we failed to observe any differences in the production of NO and ROS by MDSC from non-metastatic and metastatic patients (data not shown). CD39 and CD73 were stronger expressed on circulating M- than PMN-MDSC from melanoma patients (**Figure 2E**). While testing MDSC function using the inhibition of T cell proliferation assay, we observed that MDSC isolated from non-metastatic patients showed no immunosuppressive activity (**Figure 2F**). In contrast, metastatic patients formed two groups according to the immunosuppressive function of PMN- and M-MDSC: one group with high and another with low suppressive activity of MDSC. Interestingly, there was no difference between the immunosuppressive potential of PMN- and M-MDSC (**Figure 2F**).

**Figure 3:**
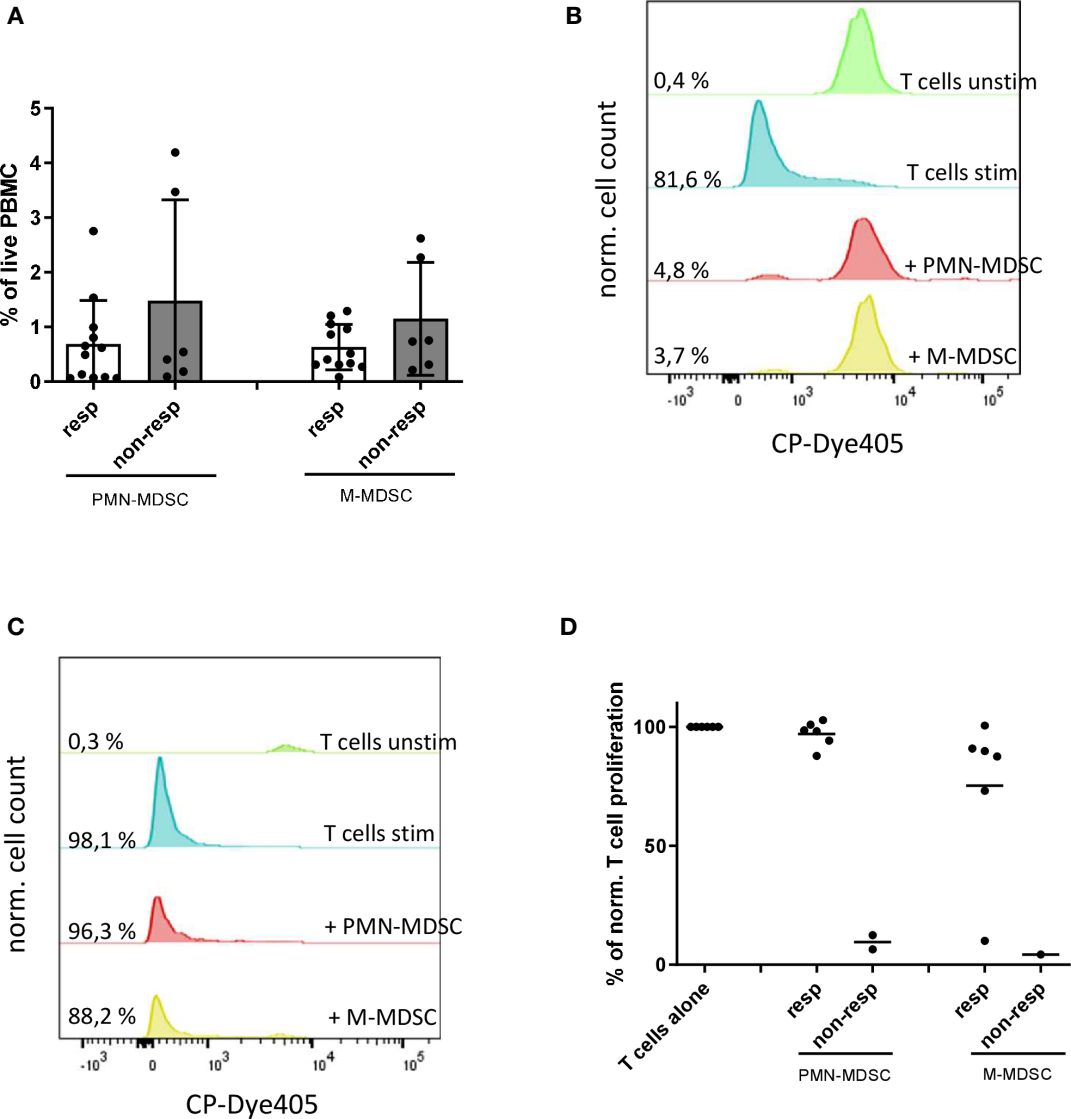
Baseline characteristics of PMN- and M-MDSC from responders and non-responders. **(A)** Results are presented as the frequency of circulating PNM- and M-MDSC among live PBMC from responders (n=12) and non-responders (n=6). Representative histograms for the proliferation of unstimulated (unstim) and stimulated (stim) T cells incubated alone or in the presence of isolated PMN- or M-MDSC from a non-responding **(B)** and responding patient **(C)**. **(D)** Immunosuppressive capacity of PMN- and M-MDSC was determined upon the co-culture with activated CD3 T cells labeled with CP-Dye405. Cumulative data for T cell proliferation are shown as the percentage of divided T cells normalized (norm.) to the respective control of stimulated T cells alone (n=2-8).

Next, we studied MDSC frequency and function at baseline in metastatic patients who responded or failed to respond to ICI. We observed a slight tendency for accumulation of PMN- and M-MDSC before the therapy start in non-responders as compared to responders (**Figure 3A**). Although we found no significant differences in the expression of immunosuppressive molecules PD-L1, CD39 and CD73 on MDSC between these two groups (**Supplementary Figure 3**), we determined a clear difference in immunosuppressive activity of these cells. PMN- and M-MDSC isolated from non-responders showed a strong inhibition of T cell proliferation (**Figures 3B, D**), whereas MDSC from responders were not immunosuppressive (**Figures 3C, D**) except one patient whose M-MDSC displayed immunosuppressive activity at the baseline but not after the 1^st^ ICI application (**Figure 3D**).

### Inflammatory factors before the start of ICI treatment

**Figure 4:**
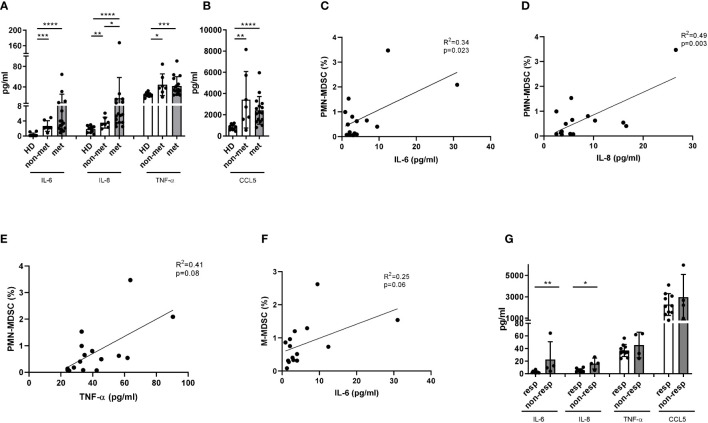
Production of inflammatory factors in melanoma patients at the baseline. Concentrations of IL-6, IL-8, TNF-α **(A)** and CCL5 **(B)** were detected in plasma of metastatic (n=16) and non-metastatic (n=7) patients as well as HD (n=10) by bio-plex assay and expressed as pg/ml. The frequency PMN-MDSC among PBMC were plotted against the level of IL-6 **(C)**, IL-8 **(D)** and TNF-α **(E)** in metastatic melanoma patients (n=15). The correlation was evaluated by a linear regression analysis. **(F)** The frequency M-MDSC within PBMC were plotted against the level of IL-6 in metastatic melanoma patients (n=15). The correlation was evaluated by a linear regression analysis. **(G)** Concentrations of IL-6, IL-8, TNF-α and CCL5 in plasma from metastatic patients, responding (n=10) and non-responding (n=4) to the ICI treatment are expressed as pg/ml. *P < 0.05, **P < 0.01, ***P < 0.001, ****P < 0.0001.

To evaluate the MDSC-related cytokine and chemokine profile in the peripheral blood of melanoma patients, we performed a bio-plex assay. We found that IL-6, IL-8, TNF-α and CCL5 were significantly increased in the plasma of metastatic and non-metastatic melanoma patients as compared to HD (**Figures 4A, B**). Moreover, metastatic patients showed significantly higher IL-8 concentrations in plasma than non-metastatic patients (**Figure 4A**). Our analysis revealed no differences in the concentration of other MDSC-related inflammatory factors (such as CCL2, CLL3, CCL4) between metastatic and non-metastatic groups (**Supplementary Figure 4A**). Interestingly, we found a correlation between increased levels of IL-6, IL-8, TNF-α and the accumulation of circulating PMN-MDSC in metastatic (**Figures 4C–E**) but not in non-metastatic patients (**Supplementary Figures 4B–D**), underlining their role in the melanoma progression. Similarly, augmented IL-6 concentration was associated with an increased frequency of M-MDSC only in patients with metastases (**Figure 4F**) but not in those without metastases (**Supplementary Figure 4E**).

To evaluate the potential of investigated soluble factors as predictive markers for the response to ICI, we compared their plasma concentrations in responders and non-responders. IL-6 and IL-8 were significantly increased at the baseline in non-responders as compared to responders, whereas TNF-α and CCL5 did not display such predictive capacity (**Figure 4G**).

### Patients with more advanced disease exhibit stronger MDSC activity

Analyzing immunosuppressive characteristics of metastatic melanoma patients before the treatment, we observed that some patients were characterized by increased MDSC frequency and immunosuppression as well as by high concentrations of MDSC-related cytokines (IL-6, IL-8). All these patients (except one who deceased before the therapy start) displayed high metastatic load and received combinational therapy with anti-CTLA-4 and anti-PD-1 antibodies. We observed that abovementioned patients with a very severe disease were also characterized by MDSC activation associated with the accumulation of IL-6 and IL-8 as well as by the non-responsiveness to combinational ICI treatment (**Table 2**).

**Table 2:** Baseline MDSC and cytokine levels in patients with metastatic melanoma who received combinational anti-PD-1 and anti-CTLA-4 treatment.

Pat.	PMN-MDSC frequency (cutoff -0.54% of live PBMC)	M-MDSC frequency (cutoff -0.73% of live PBMC)	PMN-MDSC immune suppression	M-MDSC immune suppression	IL-6 concentration (cutoff - 3.21 pg/ml)	IL-8 concentration (cutoff - 5.6 pg/ml)	Response to anti-PD-1 + anti-CTLA-4 combinational treatment	Metastases
1	high	high	+	+	high	high	Deceased before therapy begin	Liver, suprarenal gland
2	high	high	+	not determined	high	high	Non-responder	Lung, liver, brain, spleen
3	low	high	+	+	high	high	Non-responder	Lung, liver, brain, orbital cavity
4	high	low	not determined	not determined	high	high	Non-responder	Lung, liver, spinal bone, suprarenal gland
5	low	high	–	+	high	low	Responder	LN with esophagus invasion, liver

The patients were distributed in the groups with low and high frequencies of PMN- and M-MDSC as well as low and high cytokine concentrations, using their respective median values as a cutoff (19). “+” indicates the presence and “-” shows the absence of MDSC-mediated immunosuppression.

### Dynamic changes in MDSC characteristics and soluble factors under ICI treatment

**Figure 5:**
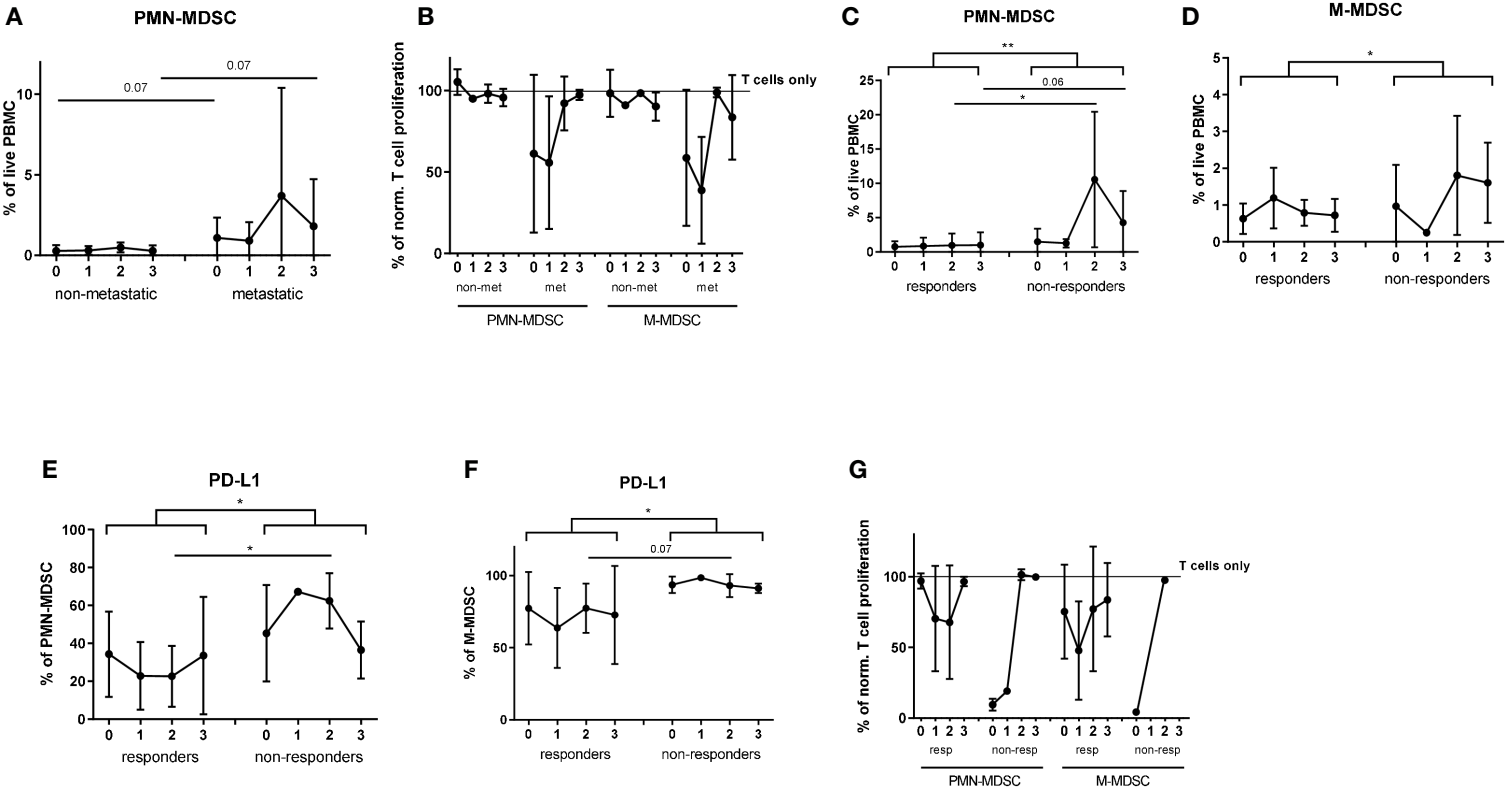
Analysis of MDSC in melanoma patients during the ICI therapy. PBMC were isolated from metastatic (n=16) and non-metastatic (n=5) patients before each ICI application (point 0 - prior the treatment; point 1 - after the first infusion; point 2 - after the second infusion; point 3 - after the third infusion) and assessed by flow cytometry. **(A)** Levels of circulating PMN-MDSC in metastatic and non-metastatic patients are expressed as the percentage within live PBMC. **(B)** Immunosuppressive capacity of PMN- and M-MDSC was determined upon the co-culture with activated CD3 T cells labeled with CP-Dye405. Cumulative data for T cell proliferation are shown as the percentage of divided T cells normalized to the respective control of stimulated T cells alone (n=5-16). Levels of circulating PMN- **(C)** and M-MDSC **(D)** in metastatic patients, responding (n=12) and non-responding (n=4) to the ICI therapy are expressed as the percentage of corresponding subsets among live PBMC. PD-L1 expression on PMN- **(E)** and M-MDSC **(F)** in responders (n=12) and non-responders (n=4) is presented as the percentage of PD-L1^+^ cells among the respective MDSC subset. **(G)** Immunosuppressive activity of PMN- and M-MDSC was measured at different time points during the ICI therapy upon the co-culture with activated CD3 T cells labeled with CP-Dye405. Cumulative data for T cell proliferation are shown as the percentage of divided T cells normalized to the respective control of stimulated T cells alone (n=1-10). *P < 0.05, **P < 0.01.

We performed the dynamic assessment of MDSC frequency and immunosuppressive function as well as MDSC-related soluble factors in metastatic and non-metastatic melanoma patients from the time point before therapy up to the first staging (baseline, after the 1^st^, 2^nd^, and 3^rd^ ICI application). We found that the frequency of PMN-MDSC in metastatic melanoma patients showed a tendency to be higher than in non-metastatic patients through the first three ICI injections (**Figure 5A**). However, there were no differences between the kinetics of M-MDSC frequency upon the therapy in metastatic and non-metastatic patients (**Supplementary Figure 5**). We also found no changes in the expression of PD-L1, CD39 and CD73 on PMN- and M-MDSC from these two groups due to high interpersonal variance (**Supplementary Figures 5B–E**). Interestingly, MDSC isolated from non-metastatic patients showed no suppressive activity towards T cells during the first three ICI applications, while metastatic patients displayed high immunosuppressive potential at baseline and after the 1^st^ ICI injection, which they tend to lose after 2^nd^ and 3^rd^ ICI application (**Figure 5B**).

Next, we analyzed the association between dynamic changes in MDSC frequency and immunosuppressive phenotype with the patients’ response to ICI. In addition to the analysis of the single time points, we compared responders and non-responders using mixed-effects model. This model allows to compare repeated measurements (before, after the 1^st^, 2^nd^, and 3^rd^ ICI application) where every patient acts as its own control and the model can handle missing values. It was found that PMN-MDSC frequency in PBMC of non-responders remained significantly higher than that in responders during first three ICI injections (**Figure 5C**). The highest frequency of PMN-MDSC was observed in non-responders after the 2^nd^ ICI injection (**Figure 5C**). Similarly, non-responders showed significantly increased M-MDSC frequency under ICI treatment as compared to responders (**Figure 5D**). Moreover, we found a significant difference in PD-L1 expression on PMN- and M-MDSC between responders and non-responders with the main difference between two groups after the 2^nd^ ICI injection (**Figures 5E, F**). Regarding ectonucleotidase expression, we found no significant differences in CD39^+^CD73^+^ MDSC between responders and non-responders (**Supplementary Figures 6A, B**).

Furthermore, we demonstrated low and nearly unchanged immunosuppressive activity of both PMN- and M-MDSC isolated from responders through three months of treatment (**Figure 5G**). In contrast, MDSC from non-responders were highly immunosuppressive at baseline and after the first injection (**Figure 5G**).

**Figure 6:**
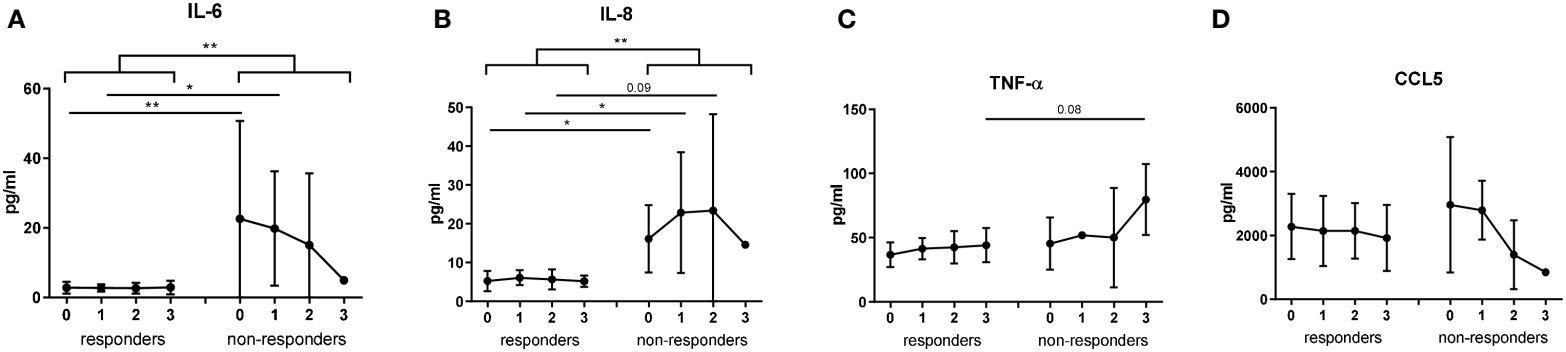
Evaluation of cytokine and chemokine concentrations in melanoma patients during the ICI treatment. Levels of IL-6 **(A)**, IL-8 **(B)**, TNF-α **(C)** and CCL5 **(D)** were measured in plasma of metastatic patients responding (n=12) and non-responding (n=4) to the ICI therapy (point 0 - before the treatment; 1 - after the first injection; 2 - after the second injection; 3 - after the third injection) by bio-plex assay and expressed as pg/ml. *P < 0.05, **P < 0.01.

Interestingly, whereas the concentration of IL-6 in responders were constantly at a very low level, its levels in non-responders tended to decrease under the ICI treatment (**Figure 6A**). Significantly elevated plasma levels of IL-6 and IL-8 at baseline and after the first ICI injection were characteristic for non-responders (**Figures 6A, B**). Importantly, the mixed-effects model revealed a significant difference in concentrations of IL-6 and IL-8 between responders and non-responders (p=0.0068 for IL-6 and p=0.0024 for IL-8); however, no association with the time point could be found (**Figures 6A, B**). In contrast, plasma levels of TNF-α and CCL5 did not significantly differ between responders and non-responders over the therapy course (**Figures 6C, D**).

## Discussion

We analyzed the frequency, immunosuppressive pattern, and function of MDSC subsets and MDSC-related soluble inflammatory mediators in the peripheral blood of melanoma patients receiving ICI. Our study provides a comprehensive analysis of dynamical changes in MDSCs phenotype and function at baseline and through three months of treatment (until the first staging) not only in advanced melanoma patients but also in those with no evidence of disease who were treated with ICI.

We observed that patients with progressive disease (in contrast to responders) tend to accumulate PMN- and M-MDSC in the peripheral blood from the baseline and through the first three months of treatment. Elevated PMN-MDSC frequency was associated with poorer OS and PFS in metastatic melanoma patients. These data are in agreement with other publications showing that elevated frequency of circulating MDSC in advanced melanoma patients correlates with decreased OS, PFS and less favorable therapy outcome (20, 21). Moreover, significantly lower PMN-MDSC amount in responding patients at the baseline as compared to non-responders was reported (22). In addition, low frequency of M-MDSC prior to the ICI treatment was previously reported to correlate with better response and increased OS (23).

Investigating the immunosuppressive capacity of MDSC, we demonstrated that circulating MDSC from non-responders exhibited the ability to inhibit T cell proliferation at baseline, whereas MDSC from responders failed to suppress T cell activity. This could be due to an enhanced T cell activation in responders (24), indicating that MDSC were not able to suppress these cells. Importantly, both PMN- and M-MDSC isolated from patients without metastases failed to inhibit T cell proliferation at any studied time point that could be explained by the deficiency of cytokines as IL-6, IL-8 and TNF-α supporting MDSC immunosuppressive function (13). Interestingly, in our study MDSC from both responders and non-responders tended to have reduced ability to suppress T cell proliferation after three doses of ICI. This could be due to the fact that anti-PD1 therapy reduces MDSC-related anergy of T cells mediated by PD1/PD-L1 interaction (25).

To decipher the mechanisms of MDSC accumulation in the peripheral blood of metastatic patients, we investigated soluble inflammatory factors involved in MDSC activation and migration like IL-6, IL-8, TNF-α and CCL5 (26). In particular, IL-6 is known to upregulate PD-L1 expression on MDSC, to lead to their activation and accumulation (27) and to cause a poor response to immunotherapy (28, 29). Moreover, IL-6 upregulates the expression of C-motif chemokine receptor (CCR) 5 on MDSC leading to their recruitment to the tumor site and enhanced inhibitory activity towards CD8^+^ T cells (30). IL-8 was shown to attract human PMN- and M-MDSC in a dose-dependent manner (31). In addition, its neutralization decreased MDSC migration (32, 33). Similarly to IL-6, high IL-8 concentration was shown to be associated with tumor progression, worse responsiveness to the ICI therapy (34) and identified as an independent biomarker of poor ICI therapy outcome (35). Furthermore, IL-8 is not only an important clinical marker of progression, but also a biomarker to monitor the clinical benefit of ICI, since early decrease in IL-8 indicated response to ICI therapy in melanoma patients and unmasked true response in cancer patients showing pseudoprogression (36). While investigating advanced melanoma patients with high tumor burden, we observed a significant increase in MDSC frequency and suppressive functions as well as in concentration of inflammatory factors in these patients, in particular IL-6 and IL-8. These findings are in line with a recent study, showing that patients with high IL-6, IL-8 concentrations and elevated MDSC frequency had worse OS (37).

Although PD-L1 expression on MDSC was reported to be significantly increased in melanoma patients with shorter PFS and worse OS (23), we did not observe such correlation in our study, which could be due to relatively low patient numbers. Interestingly, we found a tendency for the accumulation of CD39^+^CD73^+^ M-MDSC at the baseline in metastatic compared to non-metastatic patients, indicating a stronger immunosuppressive phenotype of these cells. A high expression of both CD39 and CD73 on MDSC was described to be associated with cancer progression in NSCLC (15). In addition, an increased soluble CD73 concentrations in serum of melanoma patients undergoing ICI was associated with shorter PFS and OS and was identified as an independent prognostic factor for PFS and OS in melanoma patients (38).

Our study has several limitations, including a small patient cohort and missing values at some time points that affected the power of statistical analysis. Due to a short lifespan of PMN-MDSC, we were not able to isolate these cells from each patient to perform the functional assay.

Taken together, our study highlights the role of PMN-MDSC, M-MDSC and MDSC-related inflammatory factors in melanoma progression and the outcome of ICI immunotherapy and confirms the importance of MDSC targeting together with ICI treatment on order to increase the efficiency of ICI in advanced melanoma patients. We suggest that the combination of elevated frequency and high immunosuppressive activity of circulating MDSC and increased IL-6 and IL-8 concentrations in plasma could be considered as promising prognostic biomarkers of resistance to ICI in advanced melanoma patients. These findings should be validated based on a larger patient cohort in the future.

## Data availability statement

The raw data supporting the conclusions of this article will be made available by the authors, without undue reservation.

## Ethics statement

The studies involving human participants were reviewed and approved by Das Klinische Ethikkomitee (KEK) der Universitätsmedizin Mannheim 2010-318N-MA. The patients/participants provided their written informed consent to participate in this study.

## Author contributions

VP, CG, RB, JU, and VU designed the study. VP, CG, and IA performed experiments and analyzed data. VP, RB, CG, SH, JU, and VU interpreted data and contributed to the discussion. SS, VM, and JU provided clinical expertise. VP and VU wrote the manuscript with input from all authors. All authors contributed to the article and approved the submitted version.
