# Supplementary material for: Immunosuppressive capacity of circulating MDSC predicts response to immune checkpoint inhibitors in melanoma patients
Source: Front Immunol. 2023 Feb 13;14:1065767. doi: 10.3389/fimmu.2023.1065767 (PMC9968744; doi:10.3389/fimmu.2023.1065767)
Supplement: Supplementary file 1 [file DataSheet_1.docx]

Supplementary Material


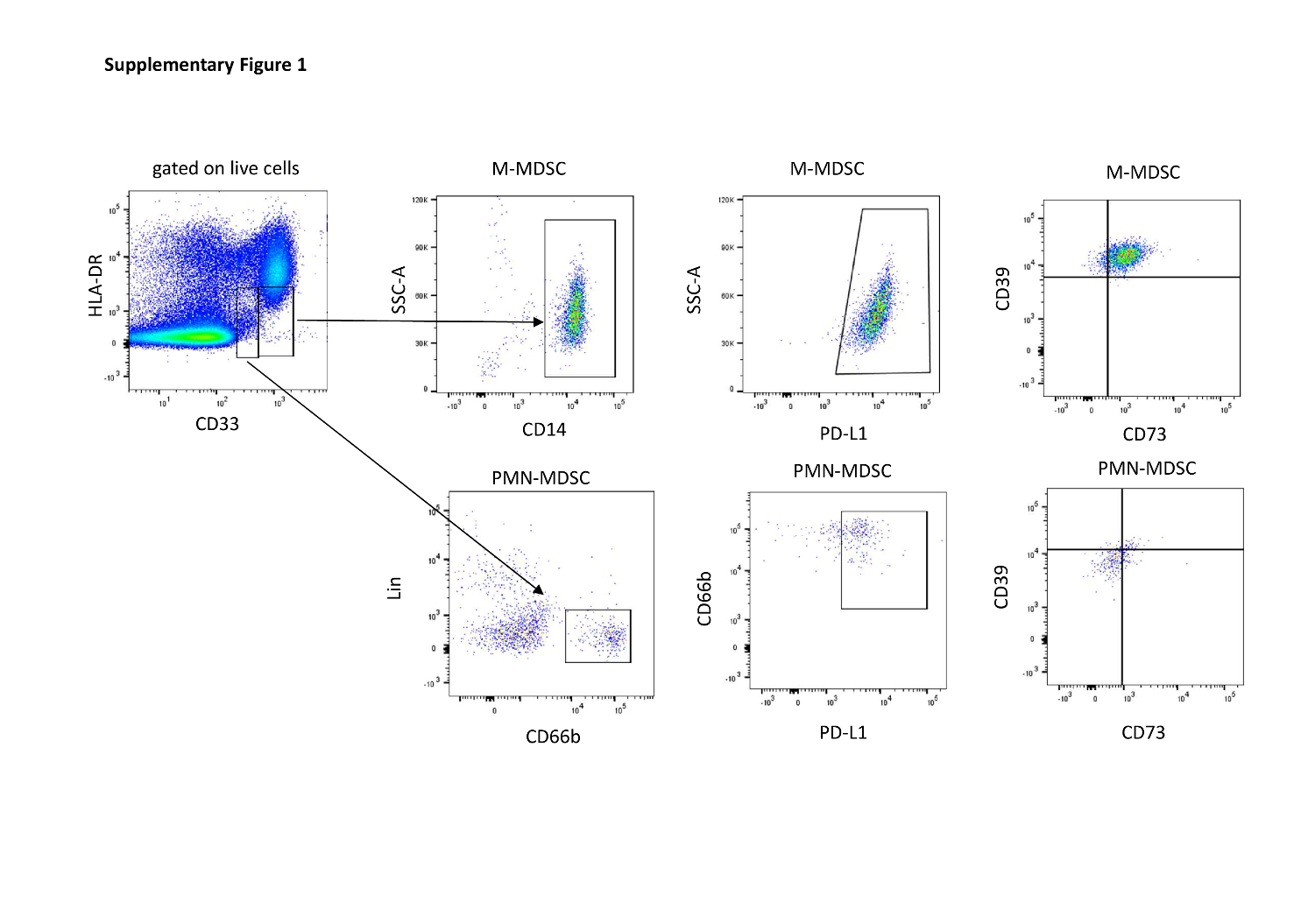


**Supplementary Figure 1.** Gating strategy for PMN- and M-MDSC from melanoma patients. Representative dot plots for HLA-DR^low/−^CD33^high^CD14^+^ M-MDSC and HLA-DR^low/−^CD33^dim^CD66b^+^Lin^−^ PMN-MDSC, expressing PD-L1, CD39 and CD73.


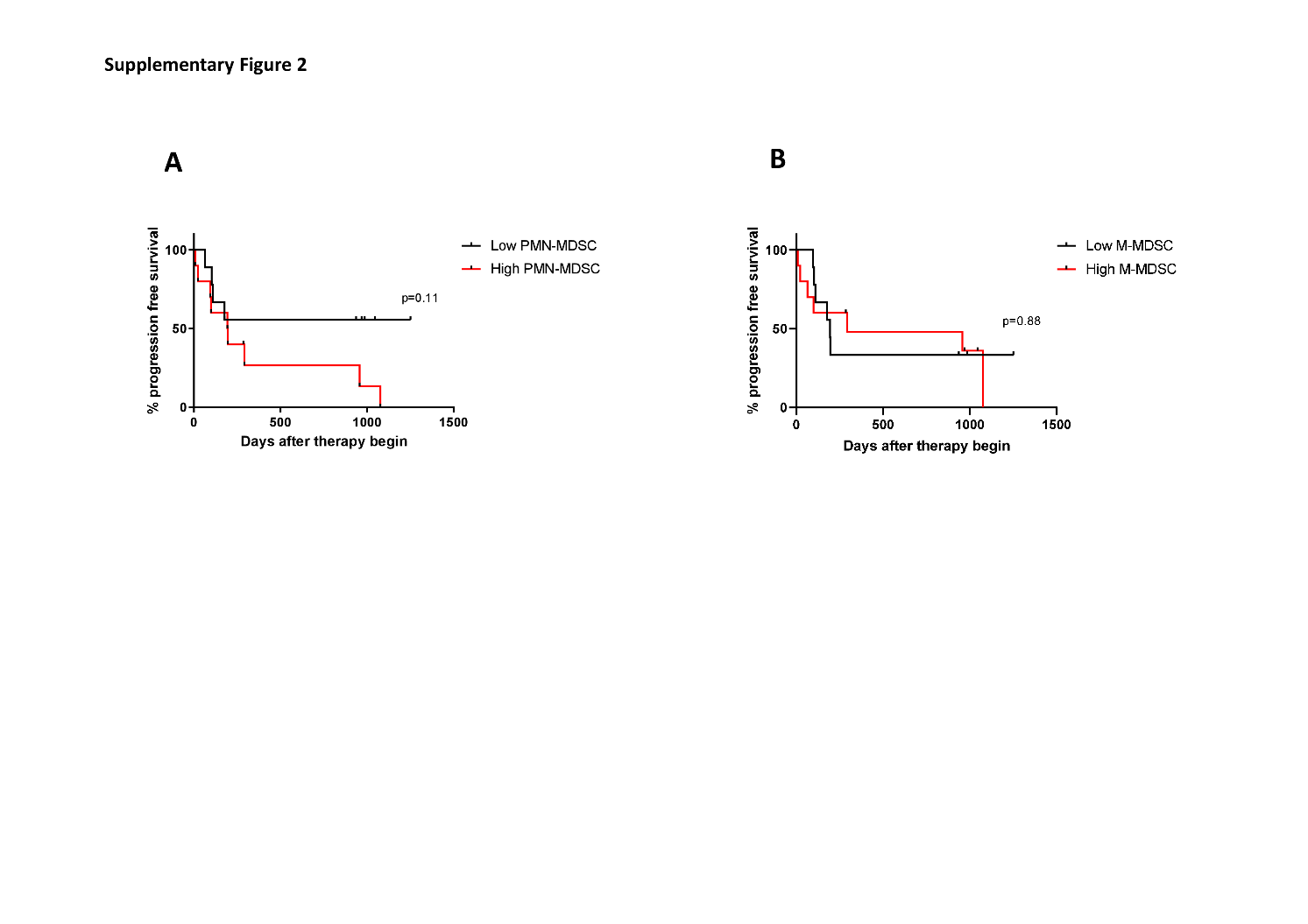


**Supplementary Figure 2.** Circulating MDSC and progression free survival (PFS) of metastatic melanoma patients. (A) PFS of patients with high (>0.54%; n=10) and low (<0.54%; n=9) PMN-MDSC frequencies at the baseline is shown as a Kaplan-Meier curve. (B) PFS of patients with high (>0,73 %: n=10) and low (<0,73 %, n=9) M-MDSC frequencies at the baseline is presented as a Kaplan-Meier curve.


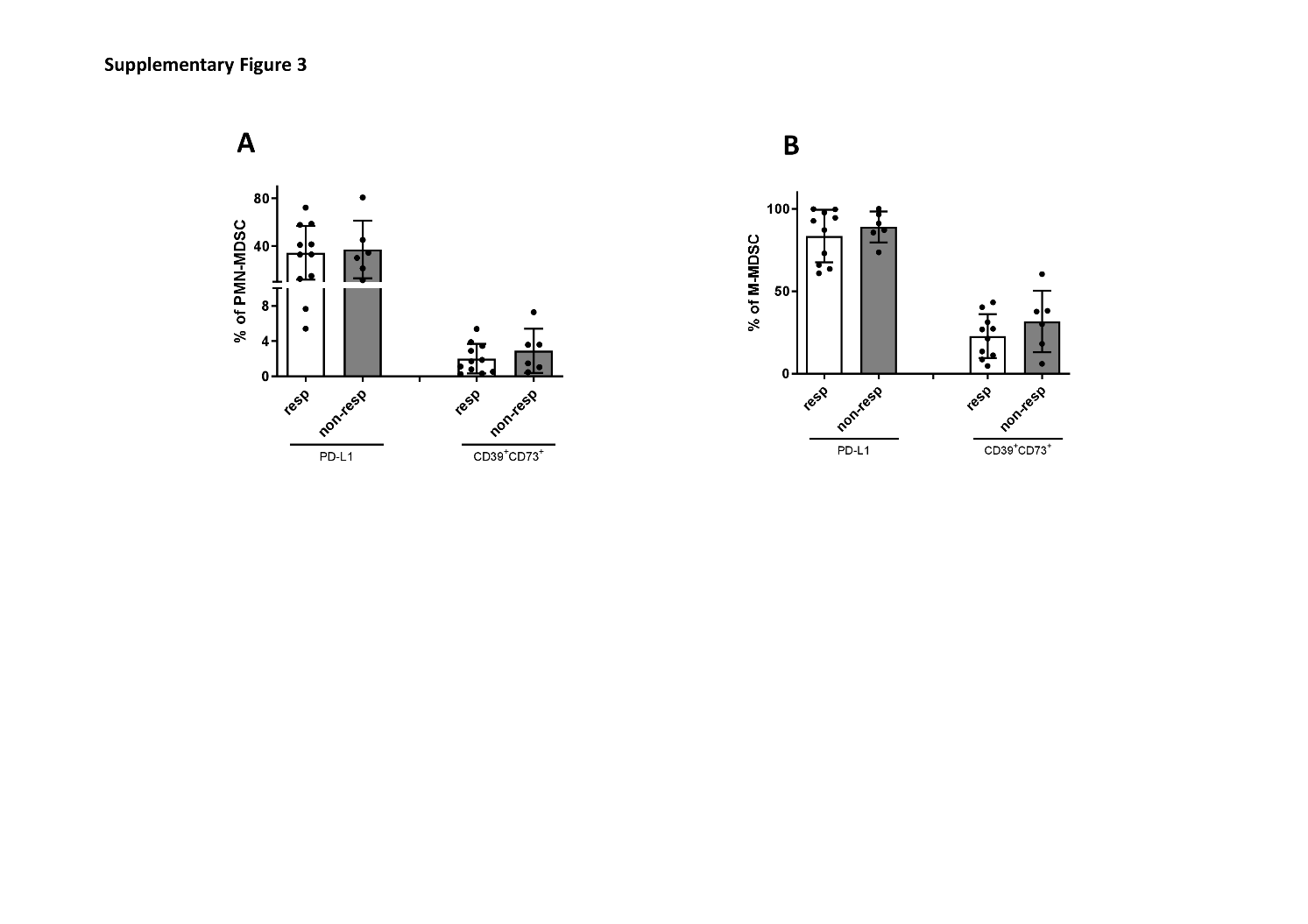


**Supplementary Figure 3.** Immunosuppressive pattern of circulating MDSC from melanoma patients during the ICI treatment. Expression of PD-L1, CD39 and CD73 was measured on PMN- (A) and M-MDSC (B) from responders (n=11) and non-responders (n=6) by flow cytometry and expressed as the frequency of PD-L1^+^ and CD39^+^CD73^+^ cells within respective MDSC subsets.


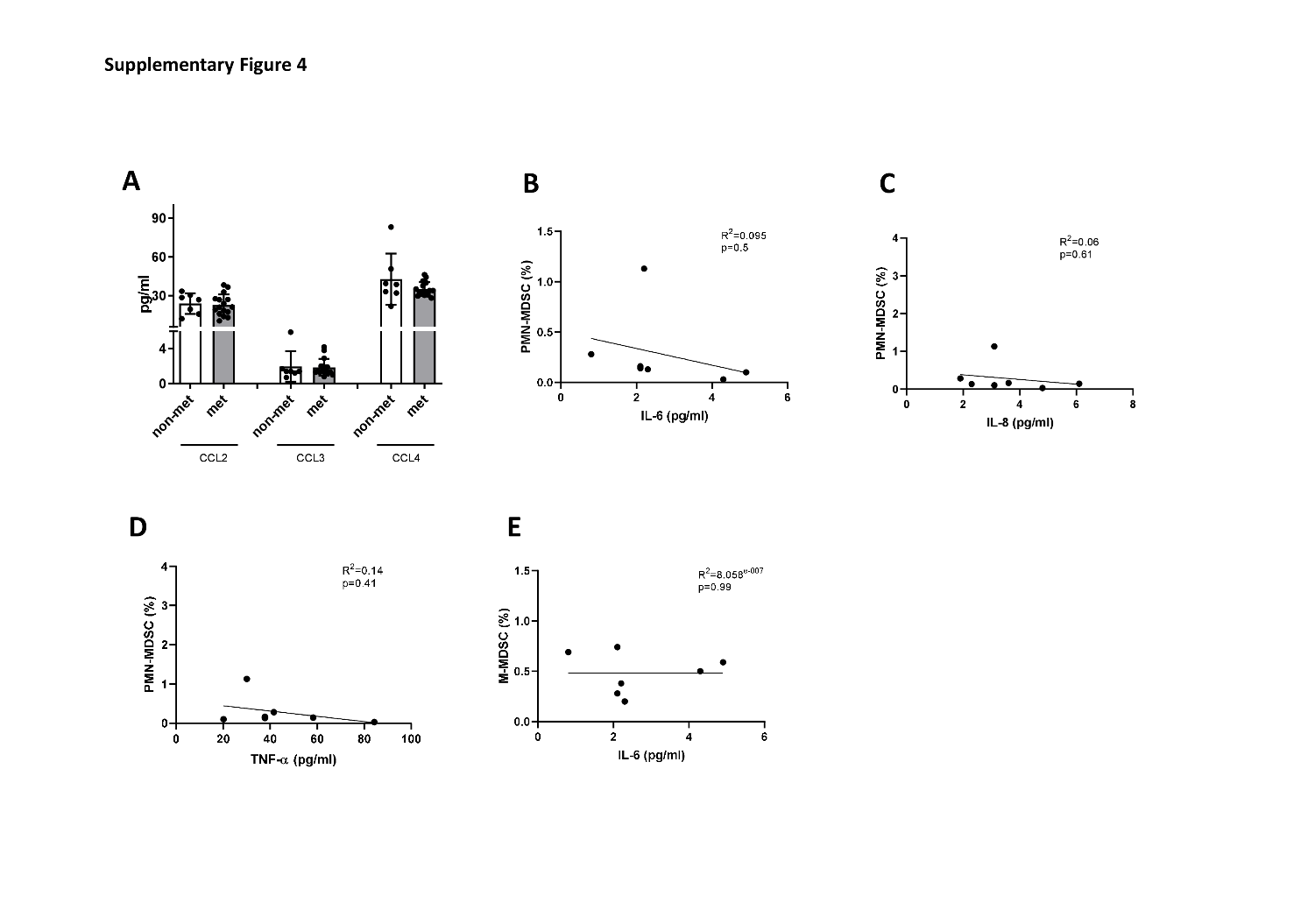


**Supplementary Figure 4.** Evaluation of inflammatory mediators and circulating MDSC in melanoma patients at the baseline. Concentrations of CCL2, CCL3 and CCL4 (A) were detected in plasma of metastatic (n=16) and non-metastatic (n=7) melanoma patients by bio-plex assay and expressed as pg/ml**.** The frequency of PMN-MDSC within PBMC were plotted against the level of IL-6 (B), IL-8 (C) and TNF- α (D) in non-metastatic melanoma patients (n=7). The frequency M-MDSC within PBMC were plotted against the level of IL-6 (E) in non-metastatic melanoma patients (n=7). The correlation was evaluated by a linear regression analysis.


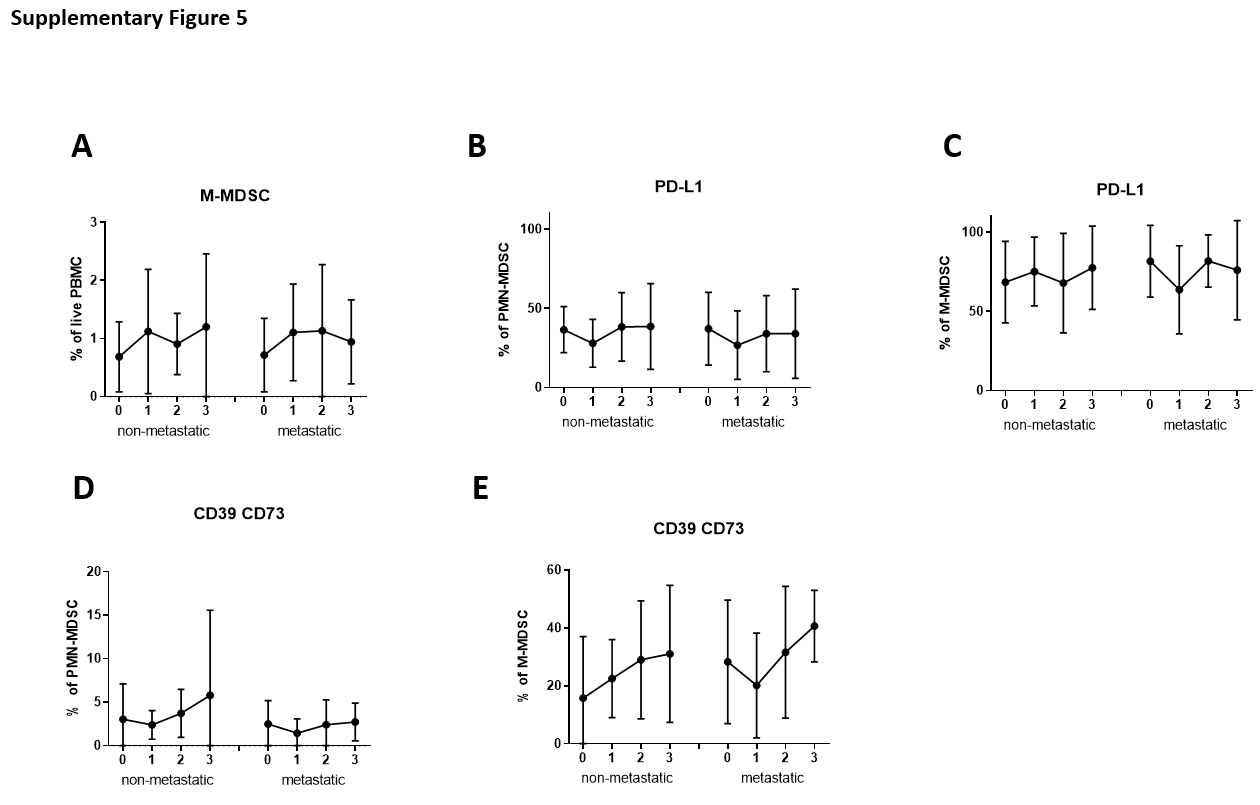


**Supplementary Figure 5.** Analysis of MDSC in melanoma patients during the ICI treatment. PBMC were isolated from metastatic (n=16) and non-metastatic (n=10) patients before each ICI application (point 0 - prior the treatment; point 1 - after the first; point 2 - after the second; point 3 - after the third injection) and evaluated by flow cytometry. (A) Levels of circulating M-MDSC in are expressed as the percentage within live PBMC. PD-L1^+^ PMN- (B) or PD-L1^+^ M-MDSC (C) are presented as the percentage among total respective subsets. CD39^+^CD73^+^ PMN- (D) or CD39^+^CD73^+^ M-MDSC (E) are presented as the percentage within respective total subsets.


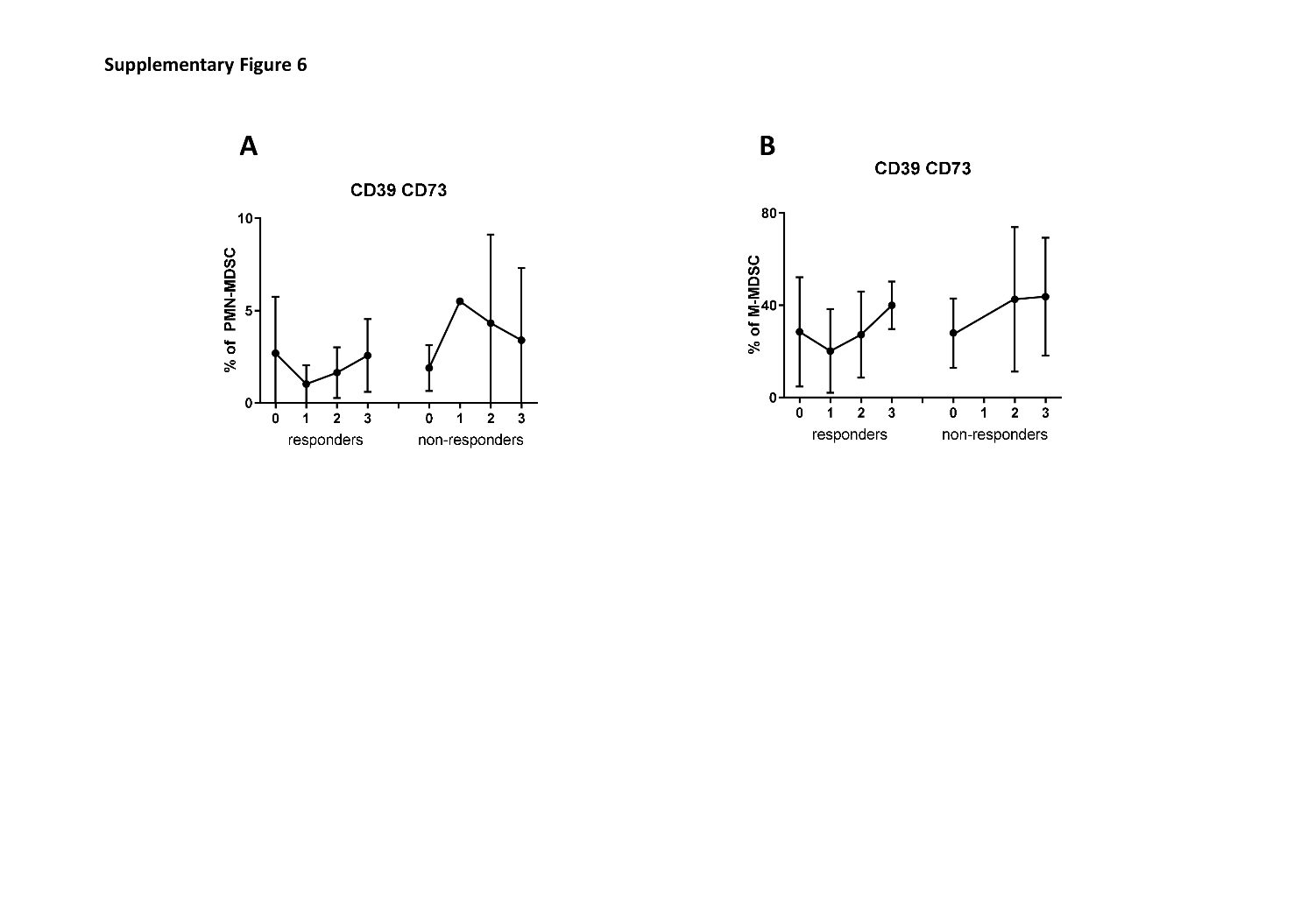


**Supplementary Figure 6.** Ectonucleotidase expression on MDSC during the ICI therapy. PBMC were isolated from patients, responding (n=12) or non-responding (n=4) to ICI (point 0 - prior the treatment; point 1 - after the first injection; point 2 - after the second injection; point 3 - after the third injection) and measured by flow cytometry. Data are expressed as the percentage of CD39^+^CD73^+^ PMN- (A) or CD39^+^CD73^+^ M-MDSC (B) within respective total MDSC subpopulations.
